# Editorial: *In vitro* transcription (IVT) reaction – the gateway to new therapeutic modalities

**DOI:** 10.3389/fmolb.2025.1730880

**Published:** 2025-11-17

**Authors:** Rok Sekirnik, Andreas N. Kuhn, Ana Margarida Azevedo, Zoltán Kis

**Affiliations:** 1 Sartorius BIA Separations d.o.o., Ajdovščina, Slovenia; 2 BioNTech SE, Mainz, Germany; 3 Instituto Superior Técncio, University of Lisbon, Lisbon, Portugal; 4 School of Chemical, Materials and Biological Engineering, University of Sheffield, Sheffield, United Kingdom; 5 Department of Chemical Engineering, Imperial College London, London, United Kingdom

**Keywords:** IVT (*in vitro* transcription), mRNA, PAT (process analytical technologies), Quality by Design (QbD), pDNA chromatography

The so-called *in vitro* transcription (IVT) reaction, the enzymatic process in which a DNA template is converted into RNA using bacteriophage RNA polymerases, has become a defining gateway to a new class of medicines. By enabling the scalable production of synthetic mRNA, IVT laid the foundation for the rise of mRNA vaccines, which received an incredible amount of public and scientific attention due to their role in mitigating the risks of the COVID-19 pandemic. Since then, mRNA technology has entered a renaissance, reflected in the steep increase in clinical trials—rising from fewer than 200 in 2021 to approximately 450 by the summer of 2025—and in the approval of the first non-COVID-19 vaccine (the RSV vaccine). Therapeutic areas have broadened to individualized cancer treatment (neoantigen therapies, Rojas et al., 2023) and gene therapies targeting genetic diseases such as cystic fibrosis (Bai et al., 2024) and sickle cell anemia (Breda et al., 2024).

mRNA manufacturing involves mRNA sequence design, synthesis, and purification for therapeutic applications. The synthesis step is performed through *in vitro* transcription of a linear DNA template, typically a linearized plasmid DNA (pDNA). This process requires the preparation of pure linearized pDNA which, in addition to the promoter required to initiate the transcription reaction, contains key sequence elements required for functional mRNA: 5′ and 3′ untranslated regions (UTRs), a coding region, and a poly(A) tail. Contrary to plasmid DNA, which is generally produced in *E. coli*, the subsequent IVT reaction is carried out in a completely cell-free environment. This reaction minimally requires the above-described DNA template, an RNA polymerase, nucleoside triphosphates (NTPs), and Mg^2+^. A 5′ cap structure on the mRNA can improve mRNA stability by protecting against exonucleases and being recognized by initiation factors to promote translation. Therefore, a 5′ cap can be added to the mRNA during the *in vitro* transcription either co-transcriptionally (“co-capping”) by adding a suitable cap-analogue to be incorporated as the initiating nucleotide building block or post-transcriptionally (“post-capping”) using a capping enzyme. Improvements in capping strategies can enhance translation efficiency and mRNA stability. Chemical modifications of the cap structure, e.g., within the triphosphate linkage, can further improve mRNA stability and enhance ribosome recruitment. The poly(A) tail length also affects both mRNA stability and translation efficiency. Increasing the tail length increases protein expression but, beyond an optimal range, translation efficiency plateaus as additional adenosine residues provide no further benefit. UTRs from various genes, including globin and genes from tobacco etch virus, are often used to enhance translation and stability.

The Research Topic “*In Vitro* Transcription (IVT) Reaction–The Gateway to New Therapeutic Modalities” brings together eight complementary contributions that collectively advance our molecular, analytical, and process-engineering understanding of IVT. These works span topics from enzyme and substrate design to real-time analytics and impurity control, reflecting the maturation of IVT from a bench-top reaction to an industrially relevant, quality-by-design-enabled manufacturing process for diverse RNA modalities, including mRNA, self-amplifying RNA (saRNA), circular RNA (circRNA), and transfer RNA (tRNA) therapeutics.

As editors, we were amazed to observe the progress reported across most of these fronts and beyond. Contributions to the Research Topic include not only the science of mRNA but also studies on DNA as a critical raw material for IVT. For example, the Research Topic published the first report on the impact of DNA template purity on the quality of IVT product (Martinez et al.), which demonstrated that impurities in a linearized DNA template can lead to production of aberrant RNA, including dsRNA, if impurities are recognized as a template by RNA polymerase. The latter received much attention. Nair and Kis examined the phylogenetic and structure-function relationship of T7 RNA polymerase and its engineered mutants designed to reduce immunogenic impurities, linking enzyme activity to the quality attributes of the mRNA product. This Research Topic was further explored by Lenk et al., who provided a comprehensive analysis of product- and process-related contaminants arising from IVT, systematically categorizing nucleotide-based impurities (such as dsRNA, abortive transcripts, and RNA:DNA hybrids) and non-nucleotide contaminants (including RNAse, endotoxins, and metal ions). Their work highlights how these impurities can activate pattern-recognition receptors and underscores the interplay between reaction design, template engineering, and purification strategy in achieving high-purity, clinically efficacious mRNA.

mRNA capping received due attention in this Research Topic, reflecting its tremendous impact on the cost-of-goods for mRNA synthesis. Kurpijewski et al. reported the synthesis of cap analogues modified at the N2 position of 7-methylguanosine and demonstrated their dual application as translation inhibitors and as capping reagents. This work is likely to spur further research into improved capping structures.

To be optimally utilized in IVT reaction, reagents including cap analogues, NTPs, and mRNA can be monitored at-line or in-line. The minireview by Lee et al. explored available analytical approaches for monitoring IVT, including light-up RNA aptamer and fluorescent dye pairs, fluorophore-labelled antisense probes, and HPLC methods. Welbourne et al. further advanced this area by developing an HPLC-based method for at-line monitoring of IVT progression, providing near-real-time information on the concentration of both building blocks (NTPs) and product (mRNA). A related analytical technique was used by Megušar et al. to investigate factors affecting transfer RNA (tRNA) synthesis, a less explored IVT-derived modality, showing that chromatographic monitoring had the potential to increase yields by at least two-fold compared to previous reports.

IVT can be performed either as a batch reaction, where all reagents are added at once, or as a fed-batch reaction, in which selected reagents are added in boluses to minimize concentrations of reagents or co-substrates that could negatively impact the yield or quality of the product or to maximize utilization of the enzyme and template. Ziegenhals et al. reported an innovative use of fed-batch strategy that maintained low steady-state concentrations of GTP and UTP with high capping and low levels of dsRNA by preventing backward transcription at the 3′ end of the DNA template.

Collectively, the contributions in this Research Topic illustrate how the IVT reaction is evolving from a simple laboratory tool into a multi-variable, data-rich biomanufacturing process. Advances in reaction monitoring (Lee et al.; Welbourne et al.; Megušar et al.), impurity mitigation (Martinez et al.; Ziegenhals et al.), enzyme and cap engineering (Nair and Kis; Kurpijewski et al.), and systems-level analysis of by-products (Lenk et al.) collectively chart the course toward predictable, high-yield, low-immunogenic RNA production. The inclusion of tRNA synthesis (Megušar et al.) broadens the scope beyond mRNA to encompass other therapeutic RNAs, foreshadowing a convergent “RNA foundry” landscape where multiple RNA species may be produced on unified IVT platforms.

This Research Topic highlights how interdisciplinary advances in enzymology, chemistry, analytics, and process engineering are transforming IVT into a controllable and automated synthesis platform for next-generation RNA therapeutics. Looking ahead, we expect that continuous innovation will focus on three interrelated frontiers: 1) polymerase engineering for enhanced fidelity, modified-nucleotide tolerance, and compatibility with new RNA architectures such as circular and self-amplifying RNA; 2) real-time digital control and automatization through soft sensors, kinetic modelling, and the use of AI in sequence design; and 3) the establishment of truly continuous mRNA production processes that integrate synthesis, capping, and purification. Together, these advances will further redefine IVT from a laboratory reaction into an enabling technology for scalable, automated, and globally accessible RNA manufacturing, paving the way for the next-generation of RNA therapeutics and decentralized production for rapid vaccine responses.
